# Supplementary material for: Integrative bioinformatics and experiments identify RIBC2 as a key regulator in the esophageal cancer
Source: PLoS One. 2026 Feb 10;21(2):e0340850. doi: 10.1371/journal.pone.0340850 (PMC12890130; doi:10.1371/journal.pone.0340850)
Supplement: S1 Table — (DOCX) [file pone.0340850.s001.docx]

|  | High risk (n = 58) | Low risk (n = 103) |
| --- | --- | --- |
| Age | 61.72 (11.87) | 62.26 (11.90) |
| Gender |  |  |
| Female | 7 (12.07%) | 16 (15.53%) |
| Male | 51 (87.93%) | 87 (84.47%) |
| Status |  |  |
| Dead | 41 (70.69%) | 23 (22.33%) |
| Alive | 17 (29.31%) | 80 (77.67%) |
| Type |  |  |
| Adenomas and Adenocarcinomas | 37 (63.79%) | 42 (40.77%) |
| Cystic, Mucinous and Serous Neoplasms | 0 (0%) | 1 (0.01%) |
| Squamous Cell Neoplasms | 21 (36.21%) | 60 (58.25%) |

**Table S1. The basic information of EC patients in low- and high-risk groups**
