# Supplementary material for: Integrative bioinformatics and experiments identify RIBC2 as a key regulator in the esophageal cancer
Source: PLoS One. 2026 Feb 10;21(2):e0340850. doi: 10.1371/journal.pone.0340850 (PMC12890130; doi:10.1371/journal.pone.0340850)
Supplement: S2 Table — (DOCX) [file pone.0340850.s002.docx]

**Table S2. 22 Prognostic related genes**

| EntrezID | Gene | HR | p.value | CI.low | CI.high |
| --- | --- | --- | --- | --- | --- |
| 3304 | HSPA1B | 1.39 | 0.0081 | 1.09 | 1.78 |
| 202374 | STK32A | 3.32 | 0.0081 | 1.37 | 8.08 |
| 388323 | GLTPD2 | 1.45 | 0.013 | 1.08 | 1.94 |
| 368 | ABCC6 | 1.65 | 0.013 | 1.11 | 2.46 |
| 11082 | ESM1 | 1.3 | 0.014 | 1.05 | 1.6 |
| 125965 | COX6B2 | 0.584 | 0.015 | 0.379 | 0.9 |
| 8862 | APLN | 1.3 | 0.017 | 1.05 | 1.61 |
| 7832 | BTG2 | 1.34 | 0.02 | 1.05 | 1.71 |
| 59283 | CACNG8 | 3.06 | 0.024 | 1.16 | 8.06 |
| 1844 | DUSP2 | 1.39 | 0.025 | 1.04 | 1.86 |
| 53820 | RIPPLY3 | 1.35 | 0.025 | 1.04 | 1.76 |
| 9353 | SLIT2 | 0.605 | 0.025 | 0.39 | 0.939 |
| 6586 | SLIT3 | 0.735 | 0.029 | 0.557 | 0.968 |
| 26150 | RIBC2 | 1.47 | 0.03 | 1.04 | 2.09 |
| 3576 | CXCL8 | 1.18 | 0.034 | 1.01 | 1.38 |
| 285800 | PRR18 | 1.59 | 0.035 | 1.03 | 2.44 |
| 3303 | HSPA1A | 1.23 | 0.04 | 1.01 | 1.5 |
| 8715 | NOL4 | 1.71 | 0.041 | 1.02 | 2.85 |
| 123041 | SLC24A4 | 0.00342 | 0.042 | 1.42e-05 | 0.819 |
| 9796 | PHYHIP | 0.515 | 0.043 | 0.271 | 0.979 |
| 390928 | ACP7 | 1.34 | 0.043 | 1.01 | 1.79 |
| 64788 | LMF1 | 0.571 | 0.046 | 0.329 | 0.989 |
