# Supplementary material for: Integrative bioinformatics and experiments identify RIBC2 as a key regulator in the esophageal cancer
Source: PLoS One. 2026 Feb 10;21(2):e0340850. doi: 10.1371/journal.pone.0340850 (PMC12890130; doi:10.1371/journal.pone.0340850)
Supplement: S3 Table — (DOCX) [file pone.0340850.s003.docx]

**Table S3. Correlation between RIBC2 expression and clinicopathological parameters of EC patients.**

| Characteristic | n | RIBC2 Level | | p-Value |
| --- | --- | --- | --- | --- |
|  |  | Low (n = 25) | High (n = 25) |  |
| Gender |  |  |  |  |
| Male | 47 | 24 | 23 | 0.552 |
| Female | 3 | 1 | 2 |  |
| Age (years) |  |  |  |  |
| ≤60 | 15 | 4 | 11 | 0.031 |
| >60 | 35 | 21 | 14 |  |
| Tumour size (cm) |  |  |  |  |
| ＜5 | 45 | 24 | 21 | 0.157 |
| ≥5 | 15 | 1 | 4 |  |
| Lymph node metastasis |  |  |  |  |
| Negative | 28 | 16 | 12 | 0.255 |
| Positive | 22 | 9 | 13 |  |
| Tumor Stage |  |  |  |  |
| T1 + T2 | 27 | 17 | 10 | 0.047 |
| T3 + T4 | 23 | 8 | 15 |  |
| Clinical stage |  |  |  |  |
| I + II | 28 | 16 | 12 | 0.255 |
| III + IV | 22 | 9 | 13 |  |
