# Supplementary figures and images for: Integrative bioinformatics and experiments identify RIBC2 as a key regulator in the esophageal cancer
Source: PLoS One. 2026 Feb 10;21(2):e0340850. doi: 10.1371/journal.pone.0340850 (PMC12890130; doi:10.1371/journal.pone.0340850)

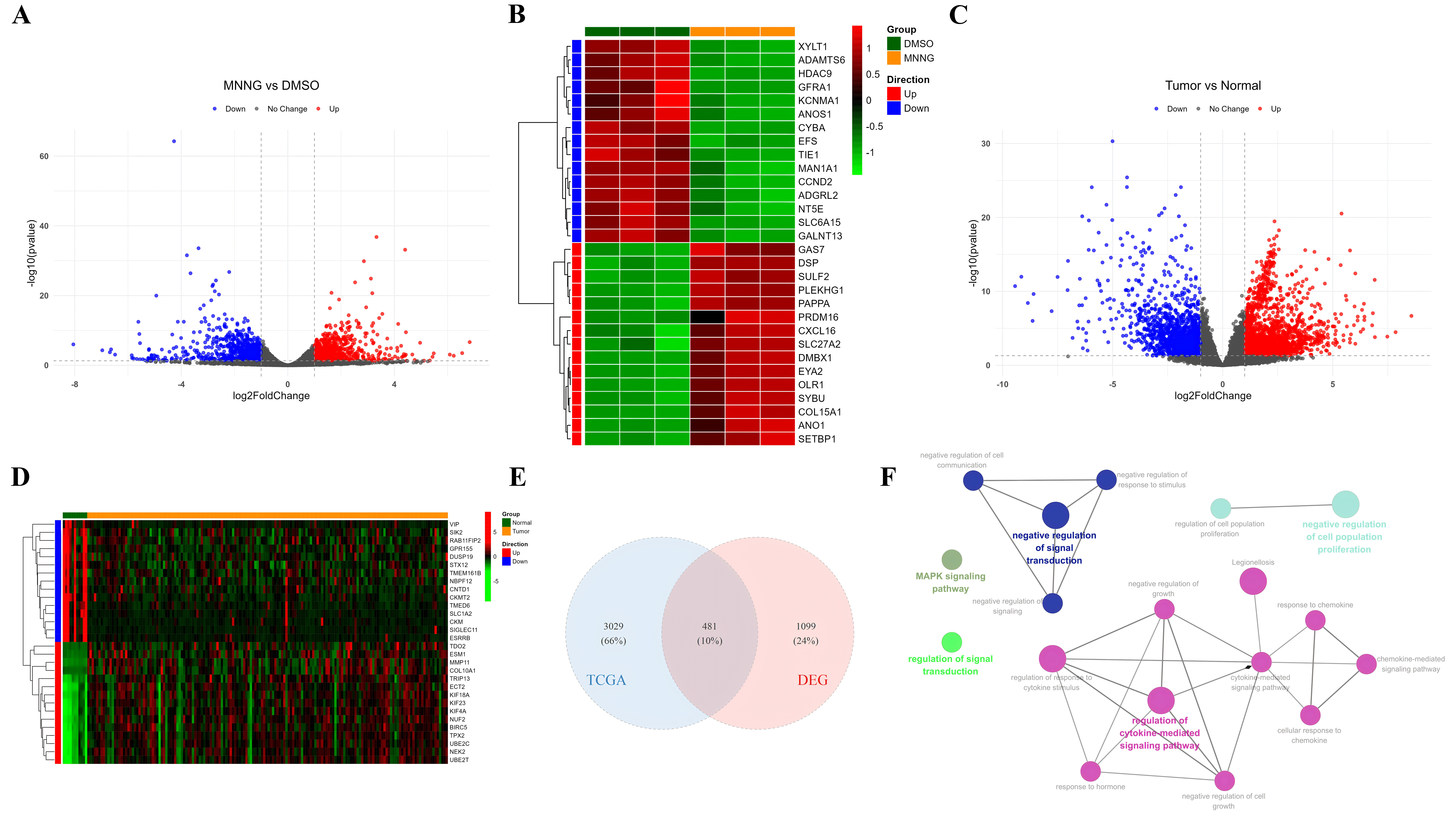

Supplement: S1 Fig — (A, B) Volcano plots and heatmap showing DEGs between Het-1A-T and Het-1A-N cells. (C, D) Volcano plots and heatmap showing DEGs between tumor and normal tissues in the TCGA-EC cohort. (E) Venn diagram showing the overlap between DEGs identified in the in vitro model and in vivo tissues. (F) Functional enrichment analysis of the 22 prognosis-related DEGs. (TIF) [file pone.0340850.s004.tif]

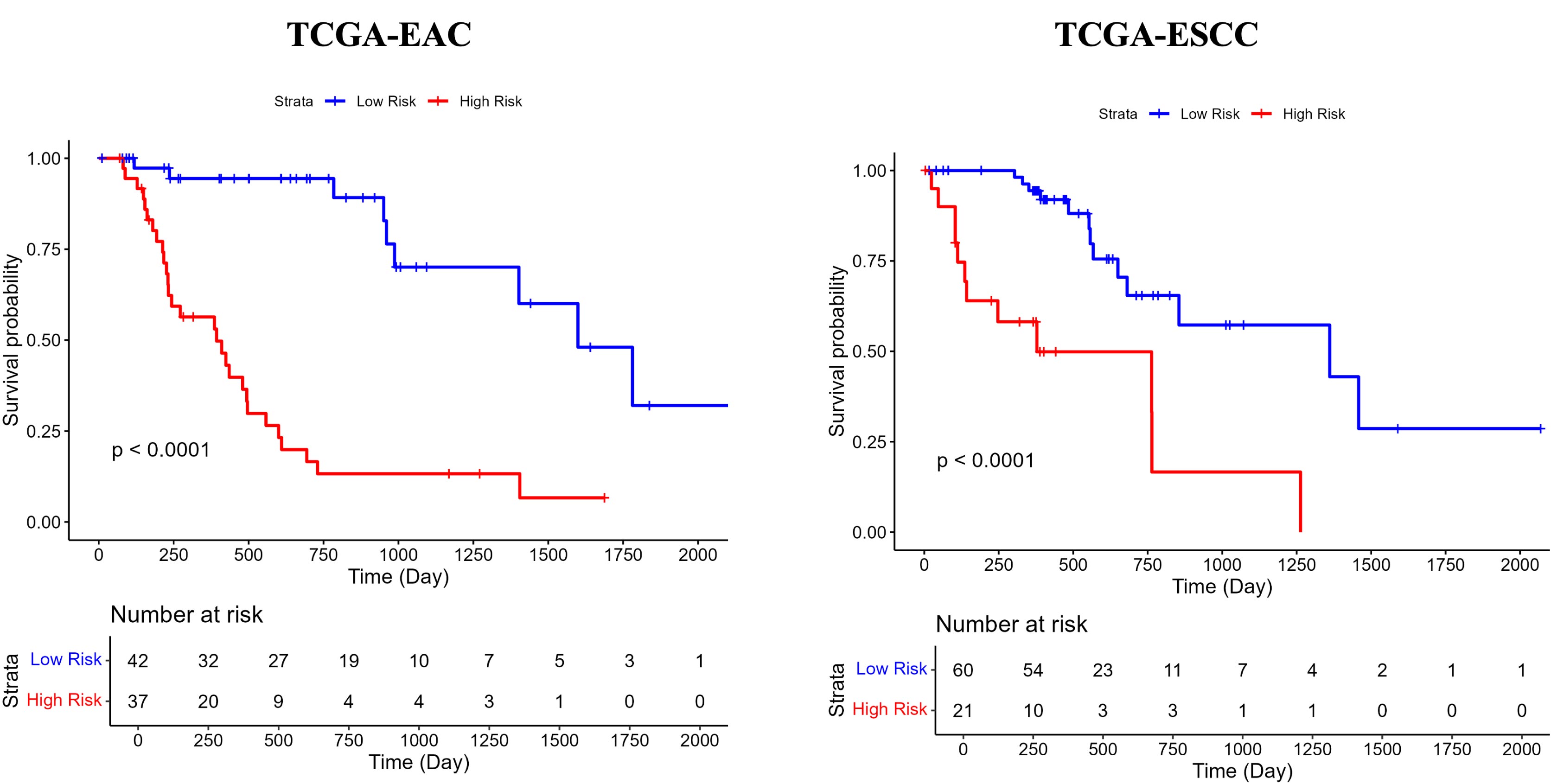

Supplement: S2 Fig — (JPG) [file pone.0340850.s005.jpg]

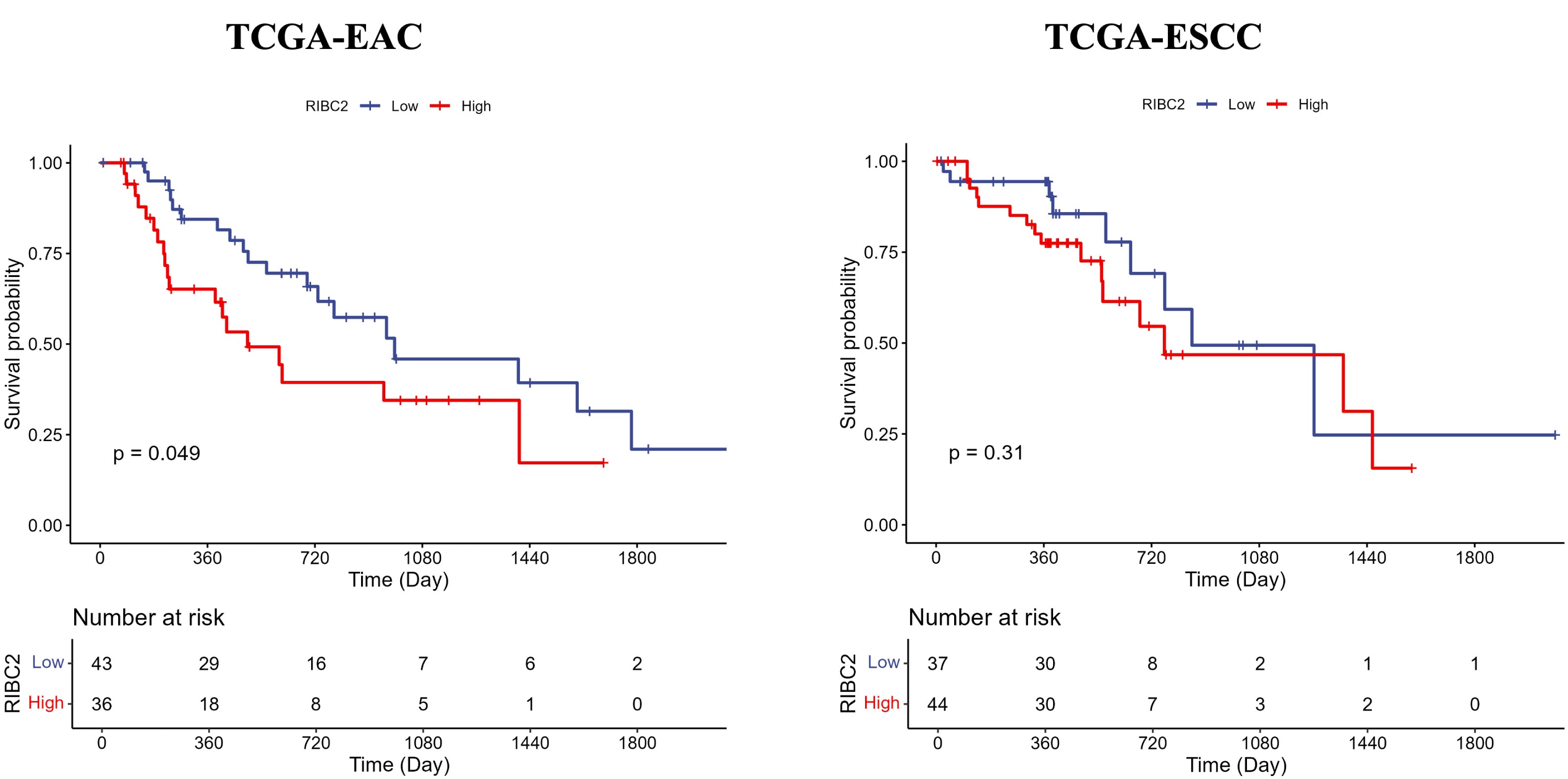

Supplement: S3 Fig — (JPG) [file pone.0340850.s006.jpg]

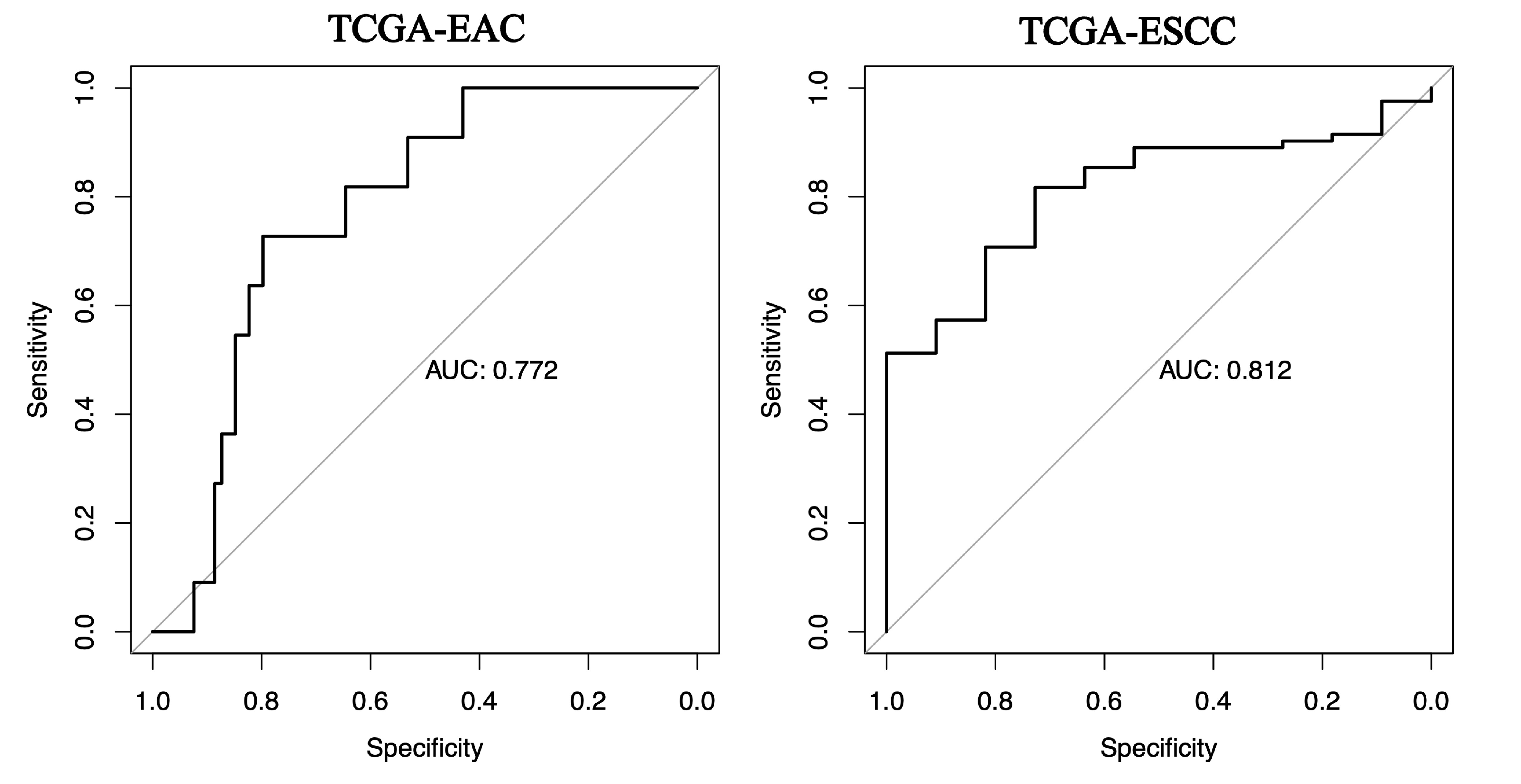

Supplement: S4 Fig — (JPG) [file pone.0340850.s007.jpg]

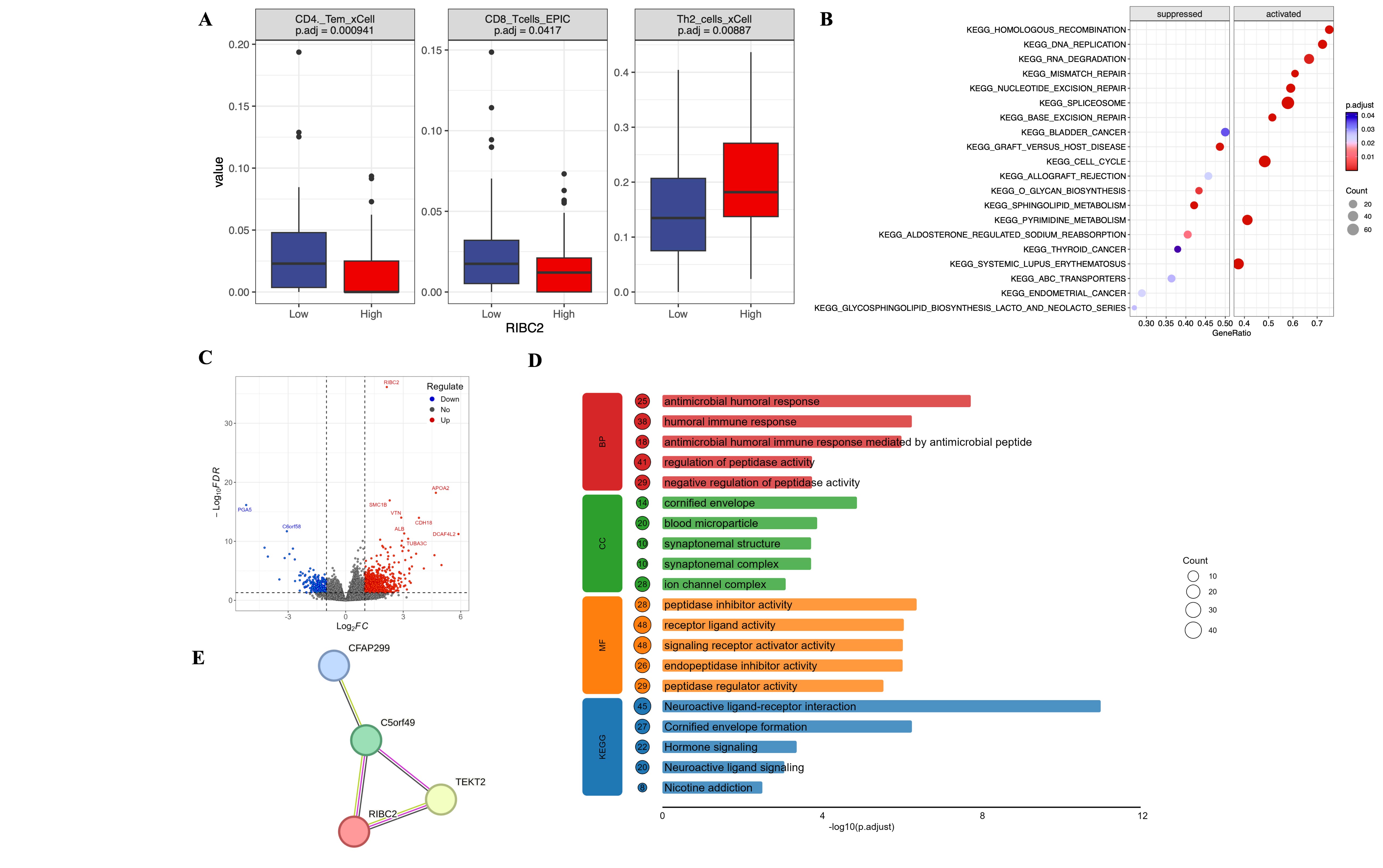

Supplement: S5 Fig — (A) Comparison of immune cell infiltration between low- and high-RIBC2 expression groups. (B) GSEA for KEGG pathways using genes ranked by the differential signal between high- and low-RIBC2 expression groups. (C) Volcano plot showing DEGs between high- and low-RIBC2 expression groups. (D) Functional enrichment analysis of DEGs between high- and low-RIBC2 expression groups. (E) PPI network highlighting RIBC2 and its candidate partners derived from STRING. Edges indicate curated or predicted interactions. (JPG) [file pone.0340850.s008.jpg]

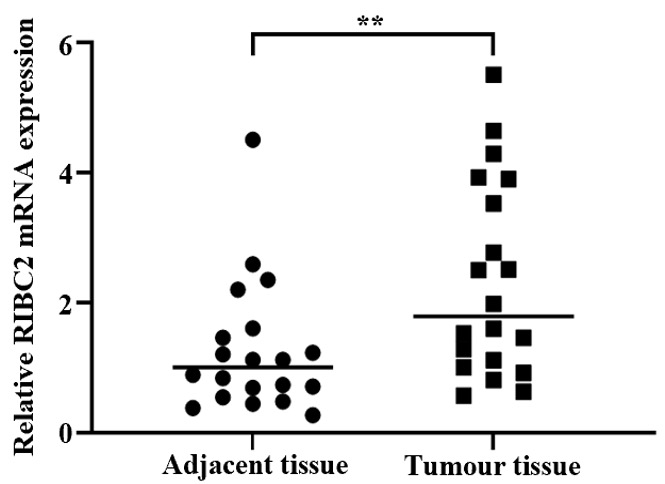

Supplement: S6 Fig — Analysis of RIBC2 mRNA expression in 20 paired EC tissues and their corresponding adjacent normal tissues. (JPG) [file pone.0340850.s009.jpg]
