# Supplementary material for: Integrative bioinformatics and experiments identify RIBC2 as a key regulator in the esophageal cancer
Source: PLoS One. 2026 Feb 10;21(2):e0340850. doi: 10.1371/journal.pone.0340850 (PMC12890130; doi:10.1371/journal.pone.0340850)
Supplement: S1 File — (PDF) [file pone.0340850.s010.pdf]

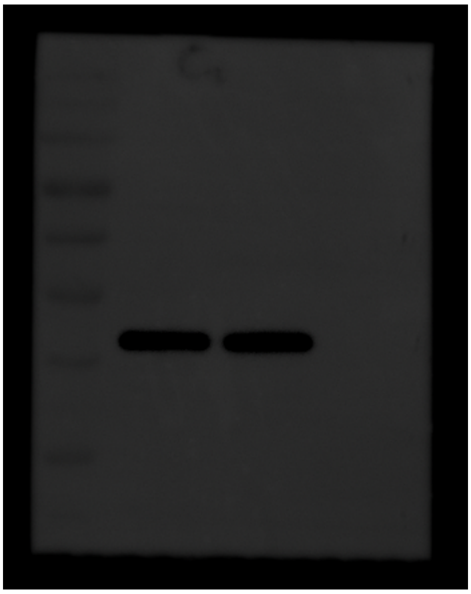

The original blot corresponding to the expression of GAPDH in Figure 5B.

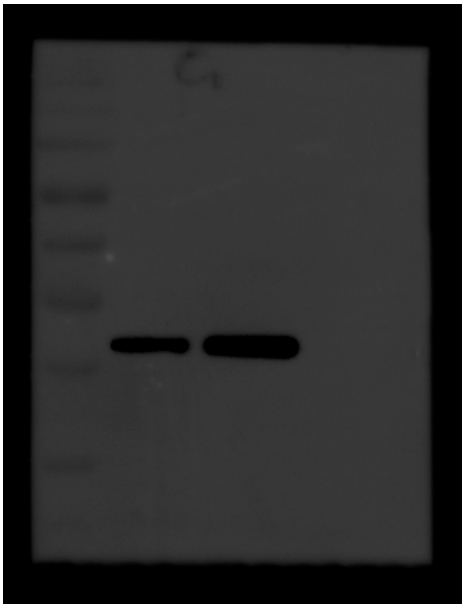

The original blot corresponding to the expression of RIBC2 in Figure 5B.

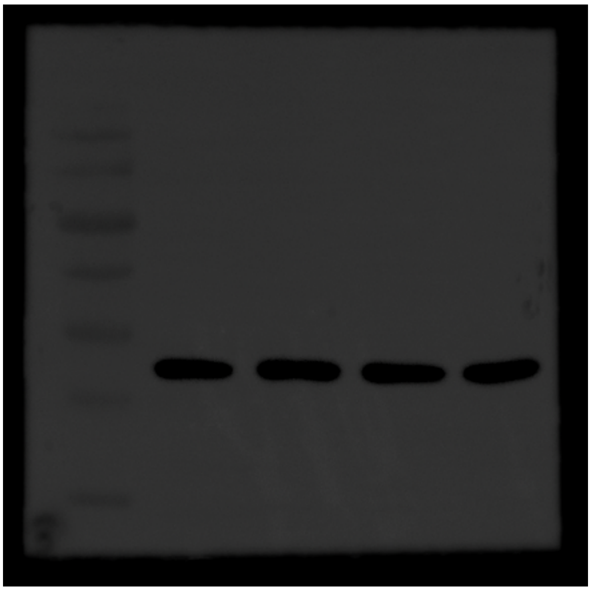

The original blot corresponding to the expression of GAPDH in Figure 5D.

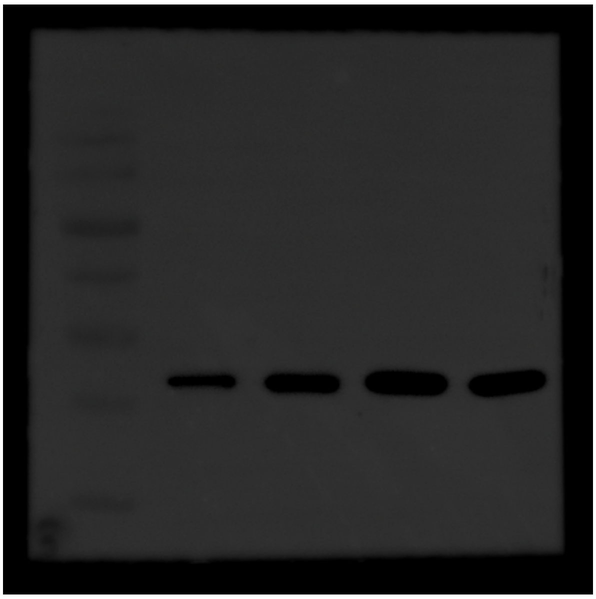

The original blot corresponding to the expression of RIBC2 in Figure 5D.

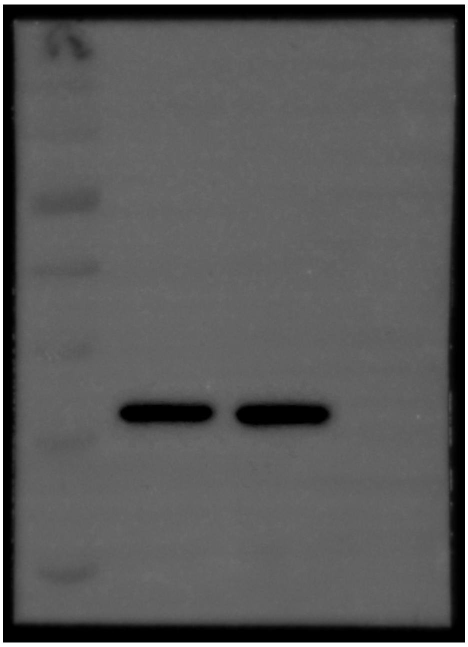

The original blot corresponding to the expression of GAPDH in Het-1A-T cells in Figure 6B.

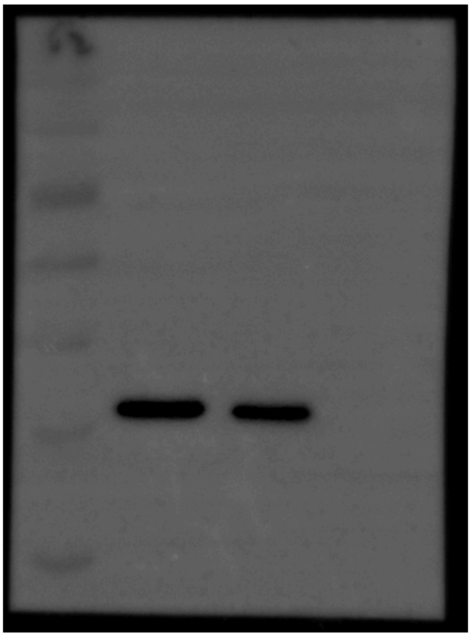

The original blot corresponding to the expression of RIBC2 in Het-1A-T cells in Figure 6B.

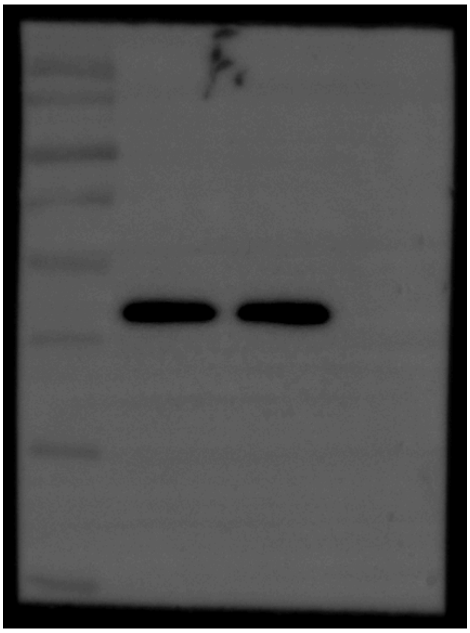

The original blot corresponding to the expression of GAPDH in OE33 cells in Figure 6B.

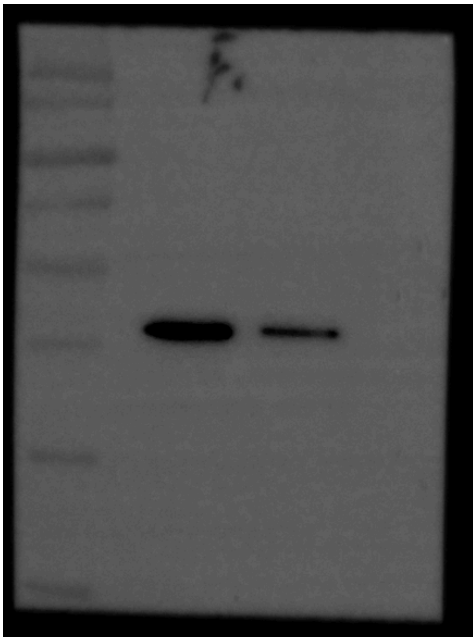

The original blot corresponding to the expression of RIBC2 in OE33 cells in Figure 6B.

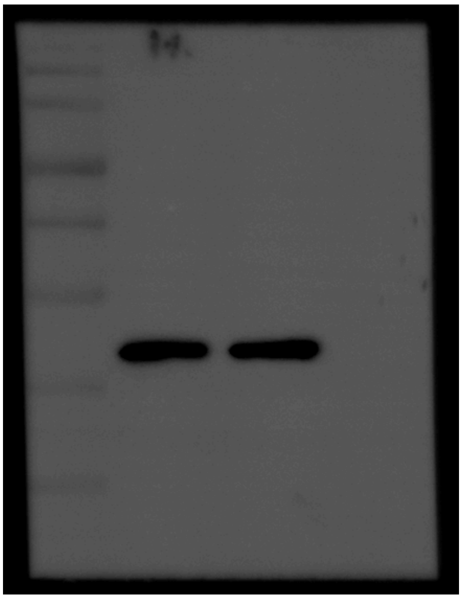

The original blot corresponding to the expression of GAPDH in TE-10 cells in Figure 6B.

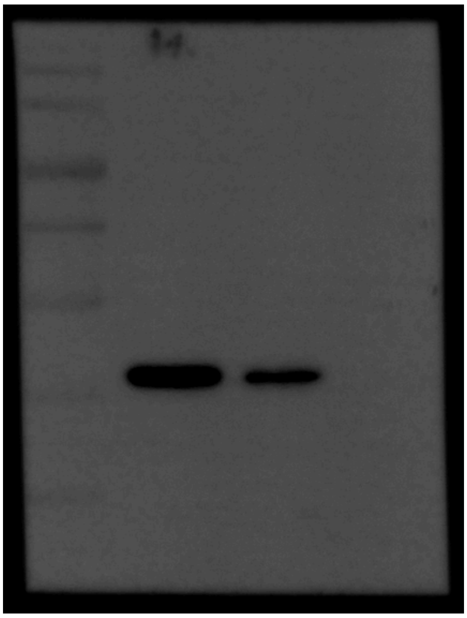

The original blot corresponding to the expression of RIBC2 in TE-10 cells in Figure 6B.
