# Supplementary material for: Integrative bioinformatics and experiments identify RIBC2 as a key regulator in the esophageal cancer
Source: PLoS One. 2026 Feb 10;21(2):e0340850. doi: 10.1371/journal.pone.0340850 (PMC12890130; doi:10.1371/journal.pone.0340850)
Supplement: Attachment — Submitted filename: Response to Reviewers.docx [file pone.0340850.s011.docx]

**Dear Editor:**

**We thank the Academic Editor and Reviewers for their thoughtful and constructive comments, which substantially improved our work. Following the suggestions, we have revised the manuscript and supporting files accordingly. Below we provide a point-by-point response, indicating where changes were made in the revised manuscript with line references.**

**#Response to Journal Requirements**

When submitting your revision, we need you to address these additional requirements.

1. Please ensure that your manuscript meets PLOS ONE's style requirements, including those for file naming. The PLOS ONE style templates can be found at

[https://journals.plos.org/plosone/s/file?id=wjVg/PLOSOne_formatting_sample_main_body.pdf](https://journals.plos.org/plosone/s/file?id=wjVg/PLOSOne_formatting_sample_main_body.pdf) and [https://journals.plos.org/plosone/s/file?id=ba62/PLOSOne_formatting_sample_title_authors_affiliations.pdf](https://track.editorialmanager.com/CL0/https:%2F%2Fjournals.plos.org%2Fplosone%2Fs%2Ffile%3Fid=ba62%2FPLOSOne_formatting_sample_title_authors_affiliations.pdf/1/010f01995567d096-e461691d-e985-420a-9fe9-0baa4f1c6d67-000000/nUObIYmvDRBRHC6mRgBhOn7YGd4zPsTO8Za5m07s3_w=228)

**Thank you for the comment. We have read the requirements carefully and revised the manuscript to conform to PLOS ONE style.**

2. To comply with PLOS One submissions requirements, in your Methods section, please provide additional information regarding the experiments involving animals and ensure you have included details on (1) methods of sacrifice, (2) methods of anesthesia and/or analgesia, and (3) efforts to alleviate suffering.

**Thank you for the comment. In accordance with PLOS ONE policies and the ARRIVE** **guidelines, we have revised the materials and methods in the revised manuscript (Lines 307-315) as “At the end of the experiment, mice were euthanized by cervical dislocation under deep anesthesia induced with sodium pentobarbital (40 mg·kg⁻¹, i.p.). Anesthetic depth was verified by the absence of pedal and palpebral reflexes. Death was confirmed by the absence of heartbeat and corneal reflex for ≥2 min. Tumors were then excised and weighed for further analysis. All animal experiments were carried out with approved protocols, relevant guidelines and regulations of the Laboratory Animal Ethics Committee of North China University of Technology (protocol code: 2023-SY-070). All methods were reported in accordance with ARRIVE guidelines.”.**

3. Thank you for stating the following financial disclosure:

[This research was funded by the National Natural Science Foundation of China, grant number 82472636; Hebei Province Innovation Capability Enhancement Plan Project, grant number 235A2403D; Hebei Provincial Department of Education Hebei Experimental Teaching and Teaching Laboratory Construction Project, grant number 81 and High level Research and Innovation Team Construction Plan of School of Public Health, North China University of Science and Technology, grant number KYTD202309.].

Please state what role the funders took in the study.  If the funders had no role, please state: "The funders had no role in study design, data collection and analysis, decision to publish, or preparation of the manuscript."

If this statement is not correct you must amend it as needed.

Please include this amended Role of Funder statement in your cover letter; we will change the online submission form on your behalf.

**Thank you for the guidance. We have added a “Role of the funders” statement. The funders had no role in study design, data collection and analysis, decision to publish, or preparation of the manuscript. The cover letter has been updated accordingly.**

4. Thank you for stating the following in your manuscript:

[This research was funded by the National Natural Science Foundation of China, grant number 82472636; Hebei Province Innovation Capability Enhancement Plan Project, grant number 235A2403D; Hebei Provincial Department of Education Hebei Experimental Teaching and Teaching Laboratory Construction Project, grant number 81 and High level Research and Innovation Team Construction Plan of School of Public Health, North China University of Science and Technology, grant number KYTD202309.]

We note that you have provided funding information that is currently declared in your Funding Statement. However, funding information should not appear in the Acknowledgments section or other areas of your manuscript. We will only publish funding information present in the Funding Statement section of the online submission form.

Please remove any funding-related text from the manuscript and let us know how you would like to update your Funding Statement. Currently, your Funding Statement reads as follows:

[This research was funded by the National Natural Science Foundation of China, grant number 82472636; Hebei Province Innovation Capability Enhancement Plan Project, grant number 235A2403D; Hebei Provincial Department of Education Hebei Experimental Teaching and Teaching Laboratory Construction Project, grant number 81 and High level Research and Innovation Team Construction Plan of School of Public Health, North China University of Science and Technology, grant number KYTD202309.]

Please include your amended statements within your cover letter; we will change the online submission form on your behalf.

**Thank you for the clarification. We have removed all funding-related text from the Acknowledgments** **and other sections of the manuscript. We will keep funding information solely in the Funding Statement per PLOS ONE policy.**

4. PLOS ONE now requires that authors provide the original uncropped and unadjusted images underlying all blot or gel results reported in a submission’s figures or Supporting Information files. This policy and the journal’s other requirements for blot/gel reporting and figure preparation are described in detail at <https://journals.plos.org/plosone/s/figures#loc-blot-and-gel-reporting-requirements> and <https://journals.plos.org/plosone/s/figures#loc-preparing-figures-from-image-files.> When you submit your revised manuscript, please ensure that your figures adhere fully to these guidelines and provide the original underlying images for all blot or gel data reported in your submission. See the following link for instructions on providing the original image data: <https://journals.plos.org/plosone/s/figures#loc-original-images-for-blots-and-gels.>

In your cover letter, please note whether your blot/gel image data are in Supporting Information or posted at a public data repository, provide the repository URL if relevant, and provide specific details as to which raw blot/gel images, if any, are not available. Email us at [plosone@plos.org](mailto:plosone@plos.org) if you have any questions.

**Thank you for the guidance. We confirm that our revised submission fully adheres to PLOS ONE blot/gel reporting requirements. We have provided the original, uncropped and unadjusted images underlying all blots, compiled as a single Supporting Information file named as “S1_File”. Also, we have included the information of original blots in the cover letter accordingly.**

5. Your ethics statement should only appear in the Methods section of your manuscript. If your ethics statement is written in any section besides the Methods, please delete it from any other section.

**Thank you for the clarification. We have removed the ethics statement from all sections other than materials and methods (Lines 113-117 and 312-315 in the revised manuscript) as “All experiments involving human tissues and clinical information were approved by the Ethics Committee of Tangshan People’s Hospital (Approval No.: RMYY-LLKS-2024042). All experiments were performed in accordance with the Declaration of Helsinki. Informed consents were obtained from all participants in the current study.” and “All animal experiments were carried out with approved protocols, relevant guidelines and regulations of the Laboratory Animal Ethics Committee of North China University of Technology (protocol code: 2023-SY-070). All methods were reported in accordance with ARRIVE guidelines.”, in accordance with PLOS ONE requirements.**

6. Please upload a new copy of Figure S1 as the detail is not clear. Please follow the link for more information: <https://blogs.plos.org/plos/2019/06/looking-good-tips-for-creating-your-plos-figures-graphics/>

**Thank you for pointing this out. We have reorganized the Figure S1 following PLOS ONE figure guidelines as shown below.**

**S1 Fig. Integrated transcriptomic analysis revealing common DEGs shared between the in vitro malignant transformation model and clinical EC tissue datasets.** (A, B) Volcano plots and heatmap showing DEGs between Het-1A-T and Het-1A-N cells. (C, D) Volcano plots and heatmap showing DEGs between tumor and normal tissues in the TCGA-EC cohort. (E) Venn diagram showing the overlap between DEGs identified in the in vitro model and in vivo tissues. (F) Functional enrichment analysis of the 22 prognosis-related DEGs.

**#Reviewer 1**

This manuscript establishes an MNNG-induced malignant transformation model of esophageal epithelial cells and combines transcriptomic sequencing with machine learning methods to identify and validate RIBC2 as a novel biomarker for esophageal cancer diagnosis and prognosis. The authors employed a systematic research strategy, starting from an in vitro malignant transformation model and integrating transcriptomic data from TCGA and GEO databases. They utilized multiple machine learning algorithms to construct diagnostic models and validated the biological significance of RIBC2 through clinical samples and functional experiments. The study found that RIBC2 is significantly overexpressed in transformed cells and esophageal cancer tissues, correlates with poor patient prognosis, and may influence immunotherapy response by regulating the immune microenvironment. However, the manuscript still requires further improvement and refinement in several aspects.

1. The authors should better elaborate in the introduction section on the important role of current multi-omics technologies in cancer biomarker discovery, particularly the application prospects of machine learning and bioinformatics methods in esophageal cancer research. With the rapid development of high-throughput sequencing and multi-omics technologies, bioinformatics has become an important tool for cancer biomarker discovery. Machine learning methods show tremendous potential in improving diagnostic accuracy and prognostic prediction, while research on immune checkpoint inhibitor-related immune toxicity also provides new perspectives for understanding the tumor immune microenvironment (doi:10.1002/imt2.70070,10.1002/mdr2.70007, PMID:37904563, 39323625).

**Thank you for the suggestion. Accordingly, we have expanded the introduction to outline the important role of multi-omics in cancer biomarker discovery, described the application prospects of machine learning and bioinformatics in the diagnosis, prognosis, therapeutic response and tumor microenvironment of EC as “With the advent of high-throughput sequencing and integrative multi-omics technologies, bioinformatics has emerged as a powerful tool for cancer biomarker discovery [3,4]. Through the systematic analysis of large-scale datasets, bioinformatic approaches enable the identification of critical molecular alterations and signaling pathways involved in tumor initiation and progression. In the case of EC, computational analyses have proven valuable for prioritizing candidate biomarkers with diagnostic, prognostic, and therapeutic potential. For instance, Ren et al. utilized transcriptomic data from the TCGA-EC cohort to identify *ANGPTL7*, *CSRP1*, *EXPH5*, *F2RL2*, *KCNMA1*, *MAGEC3*, *MAMDC2*, and *SLC4A9* as fibroblast-associated gene signatures linked to EC prognosis [5]. Similarly, Yuan et al. analyzed TCGA gene expression profiles and identified miR-205-3p, miR-452-3p, and miR-6499-3p as promising biomarkers for EC staging [6]. By high-dimensional single-cell proteomics, Han et al. built a CCR4/CCR6 chemokine-based model to stratify EC patients with different response to neoadjuvant chemoradiotherapy combined with immunotherapy [7]. The microbiomes of EC patients showed that intratumoral Streptococcus signatures could predict the response of neoadjuvant chemoradiotherapy [8]. Additionally, integrative multi-omics, combining transcriptomics, proteomics, metabolomics and microbiomics may provide complementary signals and better understanding of molecular mechanisms [9]. In EC, for example, PGK1 is found to reprogram glucose metabolism and contributes to EC progression by analyzing data of genomics, proteomics and phosphoproteomics from EC patients covering 9 histopathological stages and 3 phases [10]. Integrated analyses of genomics, epigenomics, transcriptomics and proteomics could classify EC patients into four molecular subtypes and identify 28 features in the prediction of anti-PD1 therapy response [11].**

**Machine learning provides practical tools for feature selection and model construction when analyzing multi-omics data, and may improve the diagnostic accuracy and prognostic prediction in EC. For example, HSPD1 and MAP1LC3B were identified as key prognostic genes by LASSO regression, random forest and XGBoost algorithms [12]. Li et al. established a clinic-radiomics nomogram by machine learning to predict the overall survival after definitive chemotherapy of EC patients [13]. Sun et al. employed 10 machine learning algorithms to generate 101 algorithm combinations and identified key neoantigen-related genes. These genes could stratify EC patients into groups with differential infiltration levels of dendritic cells, macrophages and B cells [14]. However, the strength of bioinformatics predictions is often constrained by the lack of clinical and experimental validation. Therefore, transforming in silico findings into clinically meaningful insights demands rigorous analytical frameworks coupled with comprehensive biological verification.” (Lines 67-90 in the revised manuscript). These relevant citations have been added accordingly (Lines 621-645 in the revised manuscript).**

2. The authors should use the IOBR package and GseaVis package for further analysis of immune infiltration and biological mechanisms (doi: 10.1002/mdr2.70000; 10.1002/mdr2.70001).

**Thank you for the suggestion. In the revision, we incorporated the IOBR R package for immune deconvolution and tumor microenvironment (TME) scoring, and used GseaVis for enhanced visualization of GSEA/GSVA results to further investigate the potential immune-related mechanisms of RIBC2. Specifically, we (i) estimated immune-cell fractions with multiple algorithms (CIBERSORT, EPIC, quanTIseq, xCell, MCP-counter) implemented in IOBR, (ii) performed pathway enrichment by GseaVis package for key pathways in the high- and low-RIBC2 expression groups. We found that the infiltration of CD4 Tem and CD8 T cells were significantly higher in the low RIBC2 expression group, while the abundance of Th2 cells were much higher in the high RIBC2 expression group. Further GSEA analysis showed that pyrimidine metabolism, cell cycle, mismatch repair and DNA replication were activated, while O-glycan biosynthesis and sphingolipid metabolism were suppressed in the high RIBC2 expression group as shown below. Accordingly, we have updated the methods as “In addition, to investigate the role of RIBC2 in tumor immune microenvironment and its potential molecular mechanisms in regulating EC, we (1) estimated the immune infiltration by IOBR package [15], (2) performed GSEA by GseaVis package [16], (3) identified the DEGs by DESeq2 using |log₂ fold change| > 1 and an adjusted p-value < 0.05, and performed functional enrichment and protein-protein interaction network in low- and high-RIBC2 expression groups.” (Lines 184-189) and results as “We also found that the infiltration of CD4 Tem and CD8 T cells were significantly higher in the low RIBC2 expression group, while the abundance of Th2 cells were much higher in the high RIBC2 expression group (Fig S2A). Further GSEA analysis showed that pyrimidine metabolism, cell cycle, mismatch repair and DNA replication were activated, while O-glycan biosynthesis and sphingolipid metabolism were suppressed in the high RIBC2 expression group (Fig S2B). Moreover, we identified a total of 1,255 DEGs between low- and high-RIBC2 expression groups (Figure S2C). These DEGs were significantly enriched into KEGG pathways of neuroactive ligand-receptor interaction, cornified envelope formation, nicotine addition, biological processes of immune response and peptidase activity (Figure S2D). Moreover, by PPI analysis, we found that RIBC2 may interact with TEKT2, C5orf49 and CFAP299 (Figure S2E).” (Lines 447-458) and cited these relevant references in the revised manuscript (Lines 646-651).**

**S2 Fig. RIBC2-associated immune context, differential expression, pathway enrichment and PPI network in EC.** (A) Comparison of immune cell infiltration between low- and high-RIBC2 expression groups. (B) GSEA for KEGG pathways using genes ranked by the differential signal between high- and low-RIBC2 expression groups. (C) Volcano plot showing DEGs between high- and low-RIBC2 expression groups. (D) Functional enrichment analysis of DEGs between high- and low-RIBC2 expression groups. (E) PPI network highlighting RIBC2 and its candidate partners derived from STRING. Edges indicate curated or predicted interactions.

3. The authors should supplement more experimental evidence regarding the functional mechanisms of RIBC2. Although the study demonstrates the effects of RIBC2 knockdown on cellular phenotypes, it lacks in-depth exploration of its specific molecular mechanisms. It is recommended to add bioinformatics analyses such as pathway enrichment analysis and protein-protein interaction network analysis, as well as related molecular mechanism validation experiments.

**Thank you for the comment. Accordingly, we performed differentially expression analysis between low- and high-RIBC2 expression groups. Also, we performed functional enrichment of DEGs and protein-protein interaction (PPI) network among RIBC2 and DEGs as shown below. We identified a total of 1,255 DEGs between low- and high-RIBC2 expression groups. These DEGs were significantly enriched into KEGG pathways of neuroactive ligand-receptor interaction, cornified envelope formation, nicotine addition, biological processes of immune response and peptidase activity. Moreover, by PPI analysis, we found that RIBC2 may interact with TEKT2, C5orf49 and CFAP299. In the revised manuscript (Lines 184-189 and 447-458), we have updated the Methods and Results. We agree that wet-lab validations would further strengthen the study. Unfortunately, we have run out of funding. Thus, we acknowledged this as a limitation and provided a future validation plan as “In addition, to validate the molecular mechanisms of RIBC2, we will confirm the putative interaction network by co-immunoprecipitation (co-IP) for candidate partners (TEKT2, C5orf49, CFAP299) in ESCC (KYSE-410, TE-10) and EAC (OE33) cells, followed by subcellular localization. Second, guided by our enrichment results, we will determine pathway-related proteins (e.g., keratinization/cornified-envelope markers such as KRT5/KRT14; neuroactive ligand–receptor nodes including nAChR subunits with downstream p-ERK/p-AKT) to evaluate whether pathway modulation attenuates RIBC2-dependent proliferation/migration/invasion.” in the revised manuscript (Lines 563-570). We hope the added results and the future plan address the reviewer’s concern and really appreciate your understanding.**

**S2 Fig. RIBC2-associated immune context, differential expression, pathway enrichment and PPI network in EC.** (A) Comparison of immune cell infiltration between low- and high-RIBC2 expression groups. (B) GSEA for KEGG pathways using genes ranked by the differential signal between high- and low-RIBC2 expression groups. (C) Volcano plot showing DEGs between high- and low-RIBC2 expression groups. (D) Functional enrichment analysis of DEGs between high- and low-RIBC2 expression groups. (E) PPI network highlighting RIBC2 and its candidate partners derived from STRING. Edges indicate curated or predicted interactions.

4. The authors could more deeply explore the role of RIBC2 in other cancer types and its potential value as a therapeutic target in the discussion section. The current discussion is relatively simple and lacks in-depth analysis of RIBC2's broad biological functions.

**Thank you for the comment. According to your suggestion, we searched published literature using RIBC2 and cancer as key words. However, so far to our knowledge, the studies on RIBC2 in cancers are few, and its biological functions remains poorly defined. The available studies have been cited in our manuscript. Thus, we have revised the discussion to give more details on the published studies of RIBC2 as “Regarding RIBC2, it has been identified as prognostic markers in liver and cervical cancers by bioinformatic analysis [38, 39]. Higher expressions of RIBC2 were associated with better survival in cervical cancer, but worse outcome in liver cancer. However, its biological function and molecular mechanisms in cancers remain unclear. It has been reported that RIBC2 is expressed in motile cilia and plays a crucial role in ciliary movement by interacting with YbX2 [40], which could modulate mRNA stability, stemness and chemoresistance in endometrial cancer [41, 42].” (Lines 537-544 in the revised manuscript).**

5. The authors should discuss in more detail the importance of multi-omics technologies in future drug development to provide a theoretical foundation for developing RIBC2 as a prognostic marker (doi: 10.1016/j.cpan.2024.12.002, 10.1016/j.cpan.2024.12.001).

**Thank you for the comment. Accordingly, we have discussed the importance of multi-omics in future drug development to guide the application of RIBC2 as a prognostic marker in EC as “Multi-omics, including genomics, epigenomics, transcriptomics, proteomics/phospho-proteomics, metabolomics, and radiomics, provides a useful tool to translate laboratory findings into therapeutically relevant biomarkers [43, 44]. Briefly, integrating multi-omics with network pharmacology can identify druggable pathways and network nodes associated with RIBC2, and develop subtype-specific strategies for patients with high versus low RIBC2 expression. Thus, in the future work, we will stratify ESCC and EAC cohorts by RIBC2 expression and perform multi-omics profiling to refine pathway signatures in the high- versus low-RIBC2 groups. Using these signatures, we will select candidate drugs via network pharmacology, followed by molecular docking against potential targets and experimental validation.” (Lines 573-583 in the revised manuscript). These relevant references have been added in the revised manuscript.**

6. The authors should carefully check the full names preceding abbreviations throughout the text to ensure that all abbreviations have their full forms when first mentioned.
**Thank you for pointing this out. We have performed a manuscript-wide audit to ensure that every abbreviation is preceded by its full term at first mention and that subsequent uses are consistent.**

7. The article requires professional native-speaker editing to reduce grammatical errors and improve idiomatic expressions.

**Thank you for this suggestion. The entire manuscript has been professionally edited by a native English-speaking scientific editor. The certificate is attached below.**

**#Reviewer 2**

The authors concluded that RIBC2 as a novel driver of EC initiation and progression, offering promise for early detection and therapeutic targeting. This manuscript seems to be informative for the readers of this journal if their constatation is scientifically true. There are many issues to be clarified before acceptance in this manuscript.

1. Please cite the source for the first sentence of the introduction. Based on Global Cancer Observatory, Esophageal cancer is the eleventh most commonly occurring cancer worldwide and it was ranked seventh in overall mortality rate in 2022.

**Thank you for the comment. Accordingly, we have cited the source (https://gco.iarc.who.int/media/globocan/factsheets/populations/900-world-fact-sheet.pdf) in the Introduction (Lines 47-48 in the revised manuscript).**

2. The histological type of the Esophageal cancer cell lines should be indicated. KYSE-410 and TE-10 are SCC, and OE33 is adenocarcinoma. The most significant issue with this paper is that it analyzes SCC and adenocarcinoma together. These two histological types differ significantly in molecular oncology, so they should be considered separately even in basic experiments. At the very least, this point should be addressed in the discussion.

**Thank you for the comment. We agree and have now explicitly indicated the histological type of each cell line:** **KYSE-410 (ESCC),** **TE-10 (ESCC),** **and** **OE33 (EAC) in the Methods as “The human EC cell lines KYSE-410 (esophageal squamous cell carcinoma, ESCC; RRID: CVCL_1352, male), TE-10 (ESCC; RRID: CVCL_1760, male), and OE33 (esophageal adenocarcinoma, EAC; RRID: CVCL_0471, female)** **were purchased from ProcellBio (Wuhan, China).**” **(Lines 120-123 in the revised manuscript). We also acknowledge that esophageal squamous cell carcinoma (ESCC)** **and** **esophageal adenocarcinoma (EAC)** **differ substantially in molecular oncology and should be considered separately. In our study,** **RIBC2 expression is elevated in both ESCC (KYSE-410, TE-10) and EAC (OE33) cells**, **and** **RIBC2 perturbation consistently alters proliferation and migration** **across these lines. These findings point to a** **shared cellular phenotype** **driven by RIBC2, yet likely** **achieved through subtype-specific signalling pathways. We have made corresponding discussions and addressed this in the revised manuscript as “We note that ESCC and EAC differ in cell of origin, risk factors and molecular architectures. Although RIBC2 knockdown produced similar phenotypes across ESCC (KYSE-410, TE-10) and EAC (OE33) cells, the underlying pathways may differ by histology. Future work using subtype-matched 3D organoids or in vivo models, together with patient-level analyses stratified by histology will be important to define subtype-specific mechanisms and clinical relevance of RIBC2.” (Lines 554-563).**

3. In RT-qPCR or western blot, it has long been argued that GAPDH is not suitable as a reference gene due to its excessive number of isoforms and high expression levels. Please comment on this.

**Thank you for your comment. We agree that the suitability of GAPDH as a qPCR reference gene or western-blot loading control is context-dependent. In our study, within our cell-line panel (KYSE-410, TE-10, OE33), the raw CT value of GAPDH in qPCR did not show appreciable variability across conditions, and it was not differentially expressed in our discovery transcriptomes comparing low- and high-RIBC2 expression groups as shown below. For Western blot, equal loading was ensured by pre-quantified total protein** **and uniform sample preparation. The uncropped membranes provided in the supplementary files show comparable GAPDH levels across different cell lines. Given these observations, along with previously reported literatures using GAPDH as reference (References), we believe our use of GAPDH is appropriate and transparent. We appreciate the reviewer’s point and will consider reference strategy in our future work.**

**References:**

**Yang R, Wan J, Ma L, Zhou F, Yang Z, Li Z, Zhang M, Ming L. TMEM44-AS1 promotes esophageal squamous cell carcinoma progression by regulating the IGF2BP2-GPX4 axis in modulating ferroptosis. Cell Death Discov. 2023 Dec 1;9(1):431.**

**Chang Z, Jia Y, Gao M, Song L, Zhang W, Zhao R, Yu D, Liu X, Li J, Qin Y. PHF5A promotes esophageal squamous cell carcinoma progression via stabilizing VEGFA. Biol Direct. 2024 Mar 1;19(1):19.**

**Talukdar FR, di Pietro M, Secrier M, Moehler M, Goepfert K, Lima SSC, Pinto LFR, Hendricks D, Parker MI, Herceg Z. Molecular landscape of esophageal cancer: implications for early detection and personalized therapy. Ann N Y Acad Sci.**

4. In Fig.1-J, background stain of IHC of Pan-CK and Ki-67 are both too strong. I must say the quality of IHC is poor.

**Thank you for your comment. According to your suggestion, we re-performed the IHC and replaced Fig. 1J as shown below. We increased the primary antibody dilutions of Pan-CK and Ki-67 to 1:4000 and 1:10000, respectively, to reduce background. Also, we’ve updated the methods (Lines 337 in the revised manuscript).**

**Figure 1. Establishment of an in vitro model of esophageal epithelial malignant transformation.** (A) Representative H&E-stained images showing distinct morphological differences between non-transformed Het-1A-N and transformed Het-1A-T cells (magnification, ×100). (B, C) Cell growth curves (B) and doubling time (C) of Het-1A-N and Het-1A-T cells. (D-G) Evaluation of malignant characteristics in Het-1A-N and Het-1A-T cells using soft agar colony formation (D, ×40), colony formation (E), wound healing (F, ×40), and Transwell invasion (G, ×100) assays. (H, I) Representative xenograft images showing tumor formation by Het-1A-T and OE33 cells in nude mice; Het-1A-N cells failed to form tumors. (J) Representative H&E and immunohistochemical staining of xenograft tumors showing Pan-CK and Ki-67 expression (magnification, ×200). *P < 0.05, **P < 0.01 and ****P < 0.0001.

5. In Fig.3-C, is there any information of these TCGA-EC cohort 161 Esophageal cancer patients? How many SCC and adenocarcinoma?

**Thank you for the comment. Accordingly, we have provided the basic information of the TCGA-EC cohort in the supporting file as shown below. Among the cohort, there are 81 ESCC and 79 EAC patients, respectively.**

|  | High risk (n = 58) | Low risk (n = 103) |
| --- | --- | --- |
| Age | 61.72 (11.87) | 62.26 (11.90) |
| Gender |  |  |
| Female | 7 (12.07%) | 16 (15.53%) |
| Male | 51 (87.93%) | 87 (84.47%) |
| Status |  |  |
| Dead | 41 (70.69%) | 23 (22.33%) |
| Alive | 17 (29.31%) | 80 (77.67%) |
| Type |  |  |
| Adenomas and Adenocarcinomas | 37 (63.79%) | 42 (40.77%) |
| Cystic, Mucinous and Serous Neoplasms | 0 (0%) | 1 (0.01%) |
| Squamous Cell Neoplasms | 21 (36.21%) | 60 (58.25%) |

6. If, as the author states, this marker is useful for the early diagnosis of esophageal cancer, then the discussion should address specifically how it could potentially be utilized.

**Thank you for the suggestion. In the revised Discussion (Lines 589-596), we’ve addressed how RIBC2 could be used for early diagnosis. First, RIBC2 can serve as a biopsy marker to aid pathologists in evaluating indeterminate dysplasia or very early lesions. Beyond tissue, minimally invasive cytology (esophageal brush/balloon) could quantify RIBC2 mRNA as part of a small RT-qPCR panel to improve detection in at-risk patients. In addition, cfRNA/EV-RNA assays may offer a non-endoscopic option for high-risk screening and post-treatment surveillance. It is worth noting that the accurate diagnostic value of RIBC2 needs validation in the real clinical world across different cohorts.**
