# Supplementary material for: Integrative bioinformatics and experiments identify RIBC2 as a key regulator in the esophageal cancer
Source: PLoS One. 2026 Feb 10;21(2):e0340850. doi: 10.1371/journal.pone.0340850 (PMC12890130; doi:10.1371/journal.pone.0340850)
Supplement: Attachment — Submitted filename: Response_to_Reviewers_auresp_2.docx [file pone.0340850.s012.docx]

**Dear Editor and Reviewers:**

**We thank the Reviewers for their thoughtful and constructive comments, which substantially improved our work. Following the suggestions, we have revised the manuscript and supporting files accordingly. Below we provide a point-by-point response, indicating where changes were made in the revised manuscript with line references.**

**#Reviewer 1**

1. The authors have satisfactorily addressed the previous queries. This version of manuscript is acceptable for the journal.

**We sincerely appreciate your positive evaluation of our revised manuscript and your recognition that the current version is acceptable for publication. Thank you again for your insightful comments and constructive suggestions in the previous round, which have greatly improved the quality and clarity of our work.**

**#Reviewer 2**

1. According to Primer Blast, the product length of the primer set of RIBC2 is 215 bp. Should the product length not be limited to 150 bp or less since mRNA extracted from FFPE is highly fragmented? Otherwise, RT-qPCR will be influenced by the degree of FFPE fragmentation rather than the actual mRNA expression levels. Please comment on this.

**We thank the reviewer for this insightful and technically important comment. We fully agree with the reviewer that, for RNA extracted from FFPE tissues, amplicon length should preferably be kept as short as possible (typically ~70-150bp) in order to minimise the impact of RNA fragmentation and crosslinking on RT-qPCR quantification. As the reviewer pointed out, longer amplicons require longer contiguous RNA templates and are therefore more sensitive to variability in RNA integrity, under such conditions, Ct values may partially reflect the degree of fragmentation rather than true transcript abundance.**

**In our original experiments, the RIBC2 primer pair indeed generated a 215 bp product, which is not optimal for FFPE-derived RNA and could potentially introduce such bias. Several factors in our design helped mitigate this risk to some extent: (1) all 50 pairs of tumour and adjacent non-tumour tissues where obtained from matched FFPE blocks and processed under the same pre-analytical conditions, thereby reducing systematic differences in RNA degradation across groups; and (2) our main analyses focused on within-pair tumour-normal comparisons rather than comparisons across unrelated specimens.**

**Nevertheless, we fully acknowledge that a shorter amplicon represents best practice. To directly address this concern, we redesigned the RIBC2 primers (F: 5’-AGGCCCTCTACACAGAGACAAG-3’, R: 5’-GCTTTTTCCTTTCCACTGACTC) to yield a shorter amplicon (147 bp, the primer blast result was shown below). Due to the limited amount of remaining RNA in some cases, we repeated the RT-qPCR analysis in a subset of 20 paired FFPE-derived tumour and adjacent non-tumour tissues from the original 50-pair cohort. The new primer pair produced a single specific melting peak and robust amplification curves. Importantly, the expression patterns of RIBC2 remained unchanged that RIBC2 mRNA levels were still significantly higher in tumour tissues than in matched adjacent tissues in this 20-pair subset as shown below. These results indicate that our RT-qPCR reflect genuine difference RIBC2 expression rather than an artefact driven by FFPE RNA fragmentation.**

**In the revised manuscript, we have (1) updated the Methods section to clarify the use of a short-amplicon RIBC2 primer pair for FFPE-derived RNA, (2) included the redesigned RIBC2 primer sequence in the Methods (Lines 207-219 in the revised manuscript), and (3) added the short-amplicon RT qPCR results from the 20 paired samples as supplementary data (Figure S6).**

**The primer blast results of RIBC2**

**The mRNA expression of RIBC2 in 20-paired tumour tissues and adjacent tissues using new the RIBC2 primer**

2. According to the provided information of TCGA-EC cohort 161 Esophageal cancer patients in Fig.3-C, the low-risk group has a higher incidence of ESCC, while the high-risk group has a higher incidence of EAC. Shouldn't the authors draw separate K-M curves for ESCC and EAC? The same applies to Fig.4-C.

**We appreciate the reviewer’s thoughtful comment on the histological heterogeneity within the TCGA-EC cohort and fully agree that oesophageal squamous cell carcinoma (ESCC) and oesophageal adenocarcinoma (EAC) should be carefully considered when evaluating prognostic markers.**

**In response, we performed additional Kaplan–Meier survival analyses stratified by histological subtype for both the risk groups defined by our 17-gene signature and for RIBC2 expression. Specifically, TCGA-EC patients were first separated into ESCC and EAC according to the TCGA annotations, and within each subtype we compared overall survival between high- and low-risk groups and between high- and low-RIBC2 expression groups.**

**For the 17-gene risk signature, high-risk patients had significantly worse survival in both ESCC and EAC as shown below. For RIBC2 expression, the adverse prognostic impact of high expression was evident in EAC, whereas in ESCC the Kaplan–Meier curves did not show a statistically significant difference between the high- and low-expression groups as shown below. Meanwhile, we also draw separate ROC curves of RIBC2 for EAC and ESCC, and found that the AUC values were 0.772 in the EAC and 0.812 in the ESCC. These findings suggest that RIBC2 is involved in the development and progression of EAC and ESCC.**

**To avoid over-complicating the main figures, we have included the subtype-stratified Kaplan–Meier curves as new supplementary figures (Fig. S2 for the risk score and Fig. S3 for RIBC2 expression) and ROC curves of RIBC2 for EAC and ESCC (Fig. S4) and have revised the Results (Lines 418-423 and 452-458 in the revised manuscript).**

**Kaplan–Meier survival analysis of the low- and high-risk groups in the TCGA-EAC and TCGA-ESCC cohorts.**

**Kaplan–Meier survival analysis of the low- and high-RIBC2 expression groups in the TCGA-EAC and TCGA-ESCC cohorts.**

**ROC curves showing the diagnostic potential of RIBC2 in the TCGA-EAC and TCGA-ESCC cohorts**

3. Experiments shown in Figure 1, Het-1-T should be ESCC. However, the authors used OE33 (EAC) as a positive control. This experimental design has problem.

**We appreciate the reviewer’s insightful comment. As the reviewer correctly points out, Het-1A is an immortalised human oesophageal squamous epithelial cell line, and its transformed counterpart Het-1A-T is expected to resemble ESCC rather than EAC. From the perspective of histological consistency, an ESCC cell line is therefore more appropriate as a positive control for the transformation and tumourigenicity experiments.**

**In our original experimental design, we indeed included the ESCC cell line KYSE-450 in the subcutaneous xenograft assays together with the EAC cell line OE33 and Het-1A-T. KYSE-450 and OE33 were used as ESCC and EAC positive controls, respectively, whereas Het-1A-N served as a negative control. These experiments consistently showed that Het-1A-T formed tumours in nude mice, with tumour growth and malignant histological features that were clearly more aggressive than Het-1A-N and comparable to established oesophageal cancer cell lines. However, in the previous version of the figure, the layout and legend did not sufficiently emphasise KYSE-450 as the ESCC positive control, which may have given the impression that our design relied mainly on OE33 as the reference.**

**To clarify this point and to better reflect the histological context, we have reorganised Figure 1 in the revised manuscript. The panels now explicitly show four groups in the subcutaneous xenograft model: KYSE-450 (ESCC positive control), OE33 (EAC positive control), Het-1A-T (transformed squamous epithelial cells) and Het-1A-N (negative control). The figure legend and the corresponding text in the Results section have been revised to clearly state the role of KYSE-450 as the ESCC positive control (Lines 376-381 and Lines 807-816 in the revised manuscript). These revisions make our experimental design more transparent and support the conclusion that Het-1A-T recapitulates key features of squamous epithelial malignant transformation and tumourigenicity in vivo.**

**Figure 1. Establishment of an in vitro model of esophageal epithelial malignant transformation.** (A) Representative H&E-stained images showing distinct morphological differences between non-transformed Het-1A-N and transformed Het-1A-T cells (magnification, ×100). (B, C) Cell growth curves (B) and doubling time (C) of Het-1A-N and Het-1A-T cells. (D-G) Evaluation of malignant characteristics in Het-1A-N and Het-1A-T cells using soft agar colony formation (D, ×40), colony formation (E), wound healing (F, ×40), and Transwell invasion (G, ×100) assays. (H) Images of nude mice bearing subcutaneous xenografts generated from KYSE-450 (ESCC positive control), OE33 (EAC positive control), Het-1A-T (transformed squamous epithelial cells) and Het-1A-N (negative control). (I) Photographs of resected xenograft tumours from KYSE-450, OE33 and Het-1A-T groups; no palpable tumours were formed by Het-1A-N cells. (J) Representative H&E staining of xenograft tumours derived from KYSE-450, OE33 and Het-1A-T cells; higher-magnification views of the boxed areas are shown in the lower panels. (K) Representative immunohistochemical staining of xenograft tumors showing Pan-CK and Ki-67 expression (magnification, ×200). *P < 0.05, **P < 0.01 and ****P < 0.0001.

4. In Figure1-I, there is an error in the notation of the excised tumor. First line is OE33, second line is Het-1-T and third line is Het-1-N.

**We thank the reviewer for carefully checking the xenograft images. In our xenograft experiments, only KYSE-450, OE33 and Het-1A-T formed palpable tumours, whereas Het-1A-N did not generate any measurable masses in nude mice. In the revised manuscript, we have corrected Figure 1I so that the excised tumours are properly labelled according to their cell-line origin (KYSE-450, OE33 and Het-1A-T). The figure legend and the corresponding text in the Results section have been updated (Lines 376-381 and Lines 807-816 in the revised manuscript) accordingly to clarify that Het-1A-N cells failed to form tumours in vivo.**
